# Evolutionary expansion of the *SnRK* gene family in the halophyte *Spartina alterniflora* and functional characterization of salt-tolerant *SaSnRK2.9*

**DOI:** 10.3389/fpls.2026.1957301

**Published:** 2026-09-23

**Authors:** Chenxi Wei, Shoukun Chen, Qin Shu, Jiahui Geng, Shihua Chen, Lei Chen, Huihui Li

**Affiliations:** 1 Key Laboratory of Plant Molecular & Developmental Biology, College of Life Sciences, Yantai University, Yantai, Shandong, China; 2 State Key Laboratory of Crop Gene Resources and Breeding, National Key Facility for Crop Gene Resources and Genetic Improvement, Institute of Crop Sciences, Chinese Academy of Agricultural Sciences, Beijing, China; 3 National Nanfan Research Institute, Chinese Academy of Agricultural Sciences (CAAS), Sanya, Hainan, China; 4 Guangxi Key Laboratory of Rice Genetics and Breeding, Rice Research Institute, Guangxi Academy of Agricultural Sciences, Nanning, Guangxi, China

**Keywords:** evolution, genome-wide identification, salt stress, SaSnRK2.9, Spartina alterniflora

## Abstract

**Introduction:**

The *SnRK* (Sucrose Non-fermenting 1-Related Protein Kinase) gene family plays critical roles in plant stress responses and growth regulation.

**Methods:**

In this study, we performed a genome-wide identification of *SnRK* genes across 49 plant species and and focused on functional characterization of key members in the halophyte *Spartina alterniflora*.

**Results:**

A total of 1192 *SnRK* genes were identified across the 49 species, showing a progressive increase from algae to angiosperms. In *S. alterniflora*, 55 *SaSnRK* genes were identified and classified into SaSnRK1 (9), SaSnRK2 (21), and SaSnRK3 (25) subfamilies. These genes are unevenly distributed across 22 chromosomes and exhibit distinct gene structures and conserved motifs. Synteny analysis revealed that segmental duplication events have predominantly driven the expansion of the *SaSnRK* family. Codon usage analysis suggested that SaSnRK2 subfamily members are under stronger selective constraints and may represent highly expressed genes. Promoter *cis*-element analysis indicated an enrichment of stress- and hormone-related regulatory motifs. Expression profiling under increasing salt concentrations revealed divergent response patterns among SaSnRK2 subfamily genes, including both constitutively upregulated and high-salinity-specific isoforms. Functional characterization of a salt-inducible *SaSnRK2* gene, *SaSnRK2.9*, demonstrated that it physically interacts with two SaPP2C proteins and, when overexpressed in *Arabidopsis*, not only promotes earlier flowering by 4–6 days but also significantly enhances salt tolerance, with transgenic lines achieving a survival rate exceeding 50% following salt stress and recovery, compared to less than 20% in wild-type plants.

**Discussion:**

These findings, obtained primarily through heterologous overexpression in *Arabidopsis*, provide mechanistic insights into how a halophytic SnRK2 kinase balances stress tolerance and developmental regulation. While direct *in vivo* evidence in *S. alterniflora* is still required, our results highlight *SaSnRK2.9* as a promising candidate gene for improving salt tolerance and agronomic traits in crops.

## 1 Introduction

Soil salinity poses a severe threat to global agricultural productivity and food security with climate change worsening the problem, large areas of arable land are becoming less suitable for conventional crops, prompting a critical rethinking of breeding strategies for stress resilience (Bousfield et al., 2024). Unable to escape adverse conditions, plants have developed intricate molecular mechanisms to detect and respond to salt stress. These mechanisms activate complex signaling cascades that regulate gene expression, ion homeostasis, and metabolism to ensure survival (Waheed et al., 2024; Zhao et al., 2020). Protein kinases lie at the center of these adaptive processes, acting as molecular switches that coordinate cellular responses to osmotic and ionic imbalances (Chen et al., 2021; Cipak, 2022).

Among these, the Sucrose Non-fermenting 1 (SNF1)-related protein kinase (SnRK) family has emerged as a central pillar of plant stress signaling and metabolic regulation (Mondal et al., 2024; Son and Park, 2023). First identified in rye and subsequently characterized across the plant kingdom, the SnRK family is broadly categorized into three subfamilies: *SnRK1*, *SnRK2*, and *SnRK3*. Each subfamily is defined by a conserved N-terminal serine/threonine kinase domains, yet they possess divergent C-terminal regulatory regions that determine their specific functions (Jamsheer et al., 2019; Mondal et al., 2024). The *SnRK1* subfamily, orthologous to yeast SNF1 and mammalian AMPK, functions as a master energy sensor, maintaining cellular homeostasis under energy-limiting conditions (Hu et al., 2024). In contrast, the plant-specific *SnRK2* and *SnRK3* subfamilies have expanded significantly, reflecting their pivotal roles in adapting to environmental challenges. SnRK2 kinases are key mediators of abscisic acid (ABA) signaling and osmotic stress responses, while *SnRK3* members, also known as CBL-interacting protein kinases (CIPKs), form a complex signaling network with calcineurin B-like (CBL) proteins to decode calcium signals triggered by various stresses (Hasan et al., 2022; Li et al., 2023).

The functional significance of *SnRK* genes in stress resilience has been extensively documented across diverse crop species. In rice, the *SnRK2* family member *OsSAPK8* acts as a positive regulator, enhancing tolerance to drought, salt, and cold stresses, while *OsSAPK2* is involved in ABA-independent osmotic stress signaling (Zhong et al., 2020). In wheat, overexpression of *TaSnRK2.9* in tobacco was shown to improve salt and drought tolerance by enhancing reactive oxygen species (ROS) scavenging, and *TaSnRK2.4* and *TaSnRK2.8* confer enhanced multi-stress tolerance in *Arabidopsis* (Feng et al., 2018; Jiang et al., 2022; Zhang et al., 2017). Research in maize has further demonstrated that *ZmSnRK1* can coordinate carbon and nitrogen metabolism via the ZmRFWD3-Opaque2 axis, while specific *ZmSnRK2* members, such as *ZmSnRK2.8* and *ZmSnRK2.11* are involved in both ABA-dependent and ABA-independent signaling pathways under stress (Li et al., 2020). Beyond the Poaceae family, SnRK2s also play pivotal roles in specialized metabolism. For instance, *AaAPK1*, an ABA-responsive SnRK2-type kinase in *Artemisia annua*, positively regulates artemisinin biosynthesis by phosphorylating the AabZIP1 transcription factor (Zhang et al., 2018, 2019). In the medicinal plant *Salvia miltiorrhiza*, *SmSnRK2.6* promotes phenolic acid and tanshinone accumulation by interacting with SmAREB1, linking stress signaling to secondary metabolite production (Liu et al., 2024). These findings collectively underscore the multifaceted roles of *SnRK* genes, positioning them as powerful targets for genetic improvement of stress tolerance and metabolic engineering in crops. Despite these advances, several key questions remain unanswered. First, while SnRK2 kinases are known to mediate ABA signaling and osmotic stress responses, the mechanistic basis for their functional divergence, particularly how certain isoforms simultaneously promote stress tolerance and growth, is not well understood. Second, most functional studies have focused on glycophytic model plants, and the regulatory strategies employed by halophytes, which thrive under extreme salinity, have been largely unexplored. The halophyte *S. alterniflora* offers a unique opportunity to address these questions, given its exceptional salt tolerance and close phylogenetic relationship with major cereals.

Further, as an exceptional model for salt tolerance, the halophyte *S. alterniflora* thrives in highly saline environments by utilizing specialized salt glands to maintain a favorable K^+^/Na^+^ ratio (Yang et al., 2024, 2023). Its close phylogenetic relationship with major cereals makes it a valuable genetic reservoir. Despite its recently sequenced genome and the identification of several stress-tolerance genes (Chen et al., 2024, 2025; Gao et al., 2023; Jiang et al., 2025; Yang et al., 2024), a comprehensive, genome-wide analysis of the *SnRK* family in *S. alterniflora* remains lacking. To address this, we systematically identified and characterized the *SaSnRK* gene family, analyzing their phylogeny, structure, and expression profiles under salt stress. Given the large size and functional redundancy of the SnRK family, experimentally validating every member is impractical. Therefore, we applied our previously established phylogenomic approach, which integrates machine learning with phylogenetic profiling using evolutionary information from 365 plant species (Gao et al., 2023), to screen a core candidate, *SaSnRK2.9*, for functional validation. By elucidating its specific mechanisms in salt stress response and developmental regulation, this study provides crucial insights into the functional divergence of the *SnRK* family and highlights valuable targets for the genetic improvement of salt-sensitive crops.

## 2 Materials and methods

### 2.1 Identification of *SnRK* genes in 49 species

To identify *SnRK* gene family members across diverse species, genome data and annotation files for 49 species were downloaded from the Phytozome database (Goodstein et al., 2012), including 5 algal species, 5 bryophyte species, 16 other monocotyledonous species, 22 dicotyledonous species, and *S. alterniflora*. First, previously reported SnRK protein sequences from rice were used as queries to search against the proteomes of these species via BLASTP with an *E*-value threshold of 1e-5 (Son and Park, 2023). Furthermore, Hidden Markov Model (HMM) profiles of SnRK conserved domains (PF00069 and PF07714) were obtained from the Pfam database, and an HMMER search was performed against the BlastP hits with an *E*-value threshold of 1e^-5^. Putative SnRK sequences identified by these methods were then subjected to domain validation using the NCBI-CDD database to confirm the presence of conserved SnRK domains. Finally, the validated SnRK members in each species were quantified and phylogenetically classified into SnRK1, SnRK2, and SnRK3 subfamilies based on their evolutionary relationships and domain architectures.

### 2.2 Physicochemical properties and phylogenetic analysis of *SaSnRK* genes

In the genome of *S. alterniflora*, a total of 55 *SaSnRK* genes were identified and classified into three subfamilies, designated *SaSnRK1.1* through *SaSnRK1.9* (9 genes), *SaSnRK2*.1 through *SaSnRK2.21* (21 genes), and *SaSnRK3.1* through *SaSnRK3.25* (25 genes). Physicochemical properties, including protein length, molecular weight (MW), theoretical isoelectric point (pI), instability index, aliphatic index, and grand average of hydropathicity (GRAVY), were predicted using the Expasy ProtParam tool (Wilkins et al., 1999). The chromosomal locations of *SaSnRK* genes were retrieved from the genome annotation file and visualized using TBtools (Chen et al., 2020). For phylogenetic analysis, full-length protein sequences were aligned using MUSCLE integrated into MEGA11 (Tamura et al., 2021). Subsequently, phylogenetic trees were constructed using the Neighbor-joining (NJ) method with 1,000 bootstrap replicates, and the final trees were visualized and annotated via the iTOL platform (Letunic and Bork, 2024).

### 2.3 Structure and motif analysis of *SaSnRK* genes

Gene structures were determined using TBtools based on the genome annotation files. Conserved protein motifs were identified using the MEME suite (Bailey et al., 2009), with the maximum number of motifs set to 15 and the optimum motif width set to 6–50 amino acids. Finally, the phylogenetic tree, conserved motifs, and gene structures were integrated and visualized collectively using TBtools.

### 2.4 Synteny analysis of *SaSnRK* genes

Syntenic relationships were analyzed using MCScanX with default parameters (Wang et al., 2012). Intraspecific synteny was analyzed within the *S. alterniflora* genome, while interspecific synteny was evaluated between *S. alterniflora* and three reference species (rice, maize, and *Arabidopsis*), utilizing genomic data retrieved from the Phytozome database (Goodstein et al., 2012). Syntenic gene pairs containing *SaSnRK* members were extracted and visualized using TBtools (Chen et al., 2020).

### 2.5 Mutation pressure analysis of *SaSnRK* genes

The coding sequences of *SaSnRK* genes were extracted from the genome annotation file. For each subfamily, the GC content at the first, second, and third codon positions (GC1, GC2, GC3), as well as the average of the first and second positions (GC12), calculated using in-house scripts. The effective number of codons (ENC) was computed to evaluate codon usage bias. Scatter plots of GC12 against GC3 and ENC against GC3 were generated using GraphPad Prism, and linear regression analysis was performed to determine the slope and R² values for each subfamily, indicating the strength of mutation pressure.

### 2.6 *Cis*-acting element analysis of *SaSnRK* genes

The *cis*-acting elements within 2-kb promoter regions upstream of the transcription start site (TSS) of *SaSnRK* gene were extracted from the *S. alterniflora* genome and submitted to the PlantCARE database for *cis*-acting element prediction. The identified elements were classified into categories associated with abiotic stress, growth and development, and hormone responses. Their distribution across the genes was visualized as a heatmap generated with TBtools (Chen et al., 2020).

### 2.7 Expression profiling of *SaSnRKs* and promoter activity analysis of *SaSnRK2.9*

To obtain a global view of *SaSnRK* gene expression under salt stress, we first retrieved our previously published transcriptome dataset (Chen et al., 2025) and visualized the log_2_-transformed expression values of all *SaSnRK* genes across different NaCl concentrations (100, 200, 300, 500, and 700 mM) relative to the control (0 mM). The genome assembly of *S. alterniflora* is available from the NCBI under BioProject PRJNA961432, and the transcriptome data used for expression profiling have been deposited at the NCBI Sequence Read Archive (SRA) under BioProject PRJNA1105679 with associated SRA accession numbers listed therein. The coding sequence of *SaSnRK2.9* is provided in **Supplementary Table S9**, and all primers used in this study are listed in **Supplementary Table S8**.

Further, to assess salt-responsive expression, four-week-old *S. alterniflora* seedlings were exposed to 0, 100, 300, 500, or 700 mM NaCl for 0, 0.5, 3, 9, 18 h. Total RNA was extracted using TRIzol reagent and reverse-transcribed using a PrimeScript RT kit (Accurate Biotechnology Co., Ltd, China). Quantitative real-time PCR (qRT-PCR) was performed on a CFX96 Real-Time system (Bio-Rad, USA) using a SYBR Green master mix (Accurate Biotechnology Co., Ltd). Relative gene expression levels were calculated by the 2^−ΔΔCT^ method, utilizing *SaU5* as the internal reference gene (Chen et al., 2024). All qRT-PCR experiments were performed with three independent biological replicates (each consisting of three technical replicates), and data are presented as mean ± standard deviation (SD).

To investigate the tissue-specific expression pattern of *SaSnRK2.9*, its promoter region was amplified and inserted into the pCAMBIA1301 vector to drive the *β-glucuronidase* (GUS) reporter gene. This analysis was conducted in transgenic Arabidopsis as a heterologous system to assess the regulatory potential of the *SaSnRK2.9* promoter. The recombinant construct was transformed into *Agrobacterium tumefaciums* strain (GV3101) and subsequently introduced into *Arabidopsis* via the floral dip method (Clough and Bent., 1998). Homozygous T3 lines were confirmed by hygromycin resistance screening and used for GUS staining. Transgenic seedlings at the 4–6 rosette leaf stage were subjected to GUS staining at 37 °C in the dark. Following decolorization with 70% ethanol to remove chlorophyll, the stained tissues were observed and photographed under a stereomicroscope (Leica M165 FC).

### 2.8 Subcellular localization of SaSnRK2.9 protein

The coding sequence of *SaSnRK2.9* without stop codon was cloned into the eGFP vector under the control of the CaMV35S promoter. The recombinant construct was then transformed into *Agrobacterium tumefaciens* GV3101. The transformed cells were collected and washed twice with infiltration buffer (10 mM MgCl_2_, 10 mM 2-Morpholinoethanesulfonic Acid (MES), 150 μM acetosyringone). The bacteria suspension was adjusted to an OD_600_ = 0.7–0.8 and incubate at 22–24 °C under weak light for 36–48 hours. Subsequently, temporary slides were prepared, and fluorescence signals were observed using a Laser Scanning Confocal Fluorescence Microscope (LSM 980).

### 2.9 Protein-protein interaction prediction and experimental validation

To predict the potential interaction network of *SaSnRK2.9*, its protein sequence was submitted to the STRING online database using default parameters. Candidate interacting partners were further refined by performing reciprocal BLAST searches against the *S. alterniflora* genome, genes exhibiting high sequence similarity and confidence scores were selected as potential interactors for subsequent validation.

For the yeast two-hybrid (Y2H) assay, the full-length coding sequences of *SaSnRK2.9* and the three candidate *PP2C* genes (*Sa_02G086000*, *Sa_08G277100*, and *Sa_06G148900*) were cloned into the pGBKT7 and pGADT7 vectors, respectively. The recombinant plasmids were co-transformed into the yeast strain Y2HGold using the PEG/LiAc method. Transformants were first selected on SD/−Leu/−Trp medium and then spotted onto high-stringency SD/−Ade/−His/−Leu/−Trp medium containing X-α-gal to test for protein interactions. The pGBKT7-53/pGADT7-T pair served as a positive control, and empty vectors were used as negative controls. Plates were incubated at 30 °C for 3–5 days, and colony growth with blue color was scored as a positive interaction.

For the luciferase complementation imaging (LCI) assay, the coding sequences of *SaSnRK2.9* and the three candidate *PP2C* genes were individually fused to the N-terminal (nLUC) or C-terminal (cLUC) halves of firefly luciferase in the pCAMBIA-nLUC/cLUC vectors under the control of the CaMV35S promoter. The constructs were transformed into *Agrobacterium* tumefaciens strain GV3101. Bacterial cultures were harvested, resuspended in infiltration buffer (10 mM MES, pH 5.6, 10 mM MgCl_2_, 150 μM acetosyringone), and adjusted to an OD_6__0__0_ of 0.7–0.8. Equal volumes of *Agrobacterium* strains carrying nLUC- and cLUC-fusion constructs were mixed and infiltrated into the abaxial side of 4-week-old *Nicotiana benthamiana* leaves. After infiltration, plants were grown at 22 °C under weak light for 48–72 h. For imaging, 1 mM D-luciferin (Gold Biotechnology) was sprayed onto the infiltrated leaf areas, and the leaves were kept in darkness for 5 min. Luminescence signals were captured using a Tanon 5200 Multi imaging system with a 10-min exposure time. Positive interactions were defined by the detection of significant luminescence, with empty vector combinations serving as negative controls.

### 2.10 Phenotyping of transgenic *Arabidopsis*

The coding sequence of *SaSnRK2.9* without stop codon was cloned into the modified pCAMBIA1301 vector under the control of the *CaMV35S* promoter. This vector is derived from the subcellular localization construct and carries an in-frame C-terminal GFP tag, such that the overexpressed protein is produced as a *SaSnRK2.9*-GFP fusion. The absence of the stop codon ensures translational fusion with the tag, which does not affect protein function or the observed phenotypes. The recombinant construct was introduced into *Agrobacterium tumefaciens* strain *GV3101* and transformed into *Arabidopsis thaliana* (Col-0) via the floral dip method. Homozygous T3 lines were selected by hygromycin resistance screening and confirmed by PCR.

#### 2.10.1 Flowering phenotyping

For flowering time analysis, days from sowing to the opening of the first flower were recorded, and rosette leaf number and rosette radius were measured at the flowering stage.

#### 2.10.2 Salt tolerance assay

For the salt tolerance assay, wild-type (WT) and homozygous transgenic lines were grown under normal conditions until the rosette leaves were fully expanded. The plants were then irrigated with 0, 100, or 200 mM NaCl solutions for two weeks. After treatment, the primary inflorescences were removed, and the rosette leaves were photographed to document the phenotypic responses. Subsequently, the plants were allowed to recover under normal watering for one week, after which survival rates were evaluated. Plants that regenerated new inflorescences were scored as survivors.

## 3 Results

### 3.1 Identification of *SnRK* genes in 49 different species

To comprehensively understand the evolutionary dynamics of the *SnRK* gene family, a genome-wide identification was performed across 49 species. A total of 1192 *SnRK* genes were identified (**Supplementary Table S1**), comprising 186 *SnRK1*, 409 *SnRK2*, and 597 *SnRK3* genes. Notably, in algal species such as *Ostreococcus lucimarinus*, *Micromonas spRCC299*, and *Porphyra umbilicalis*, only one or no *SnRK* gene was detected, whereas a gradual increase in gene number was observed in the subsequently evolved species *Botryococcus braunii* and *Chlamydomonas reinhardtii*. Although some plant lineages appeared to exhibit a relatively stable gene number, a significant overall expansion in *SnRK* gene copy number was observed during plant evolution. Specifically, *SnRK* gene numbers steadily increased in bryophytes and dicotyledons, whereas monocotyledons displayed considerable variation (**Figure 1**). This pattern implies that *SnRK* genes have played a crucial role in plant adaptation to diverse environments, with gene duplication serving as a strategy to cope with complex and changing conditions.

**Figure 1:**
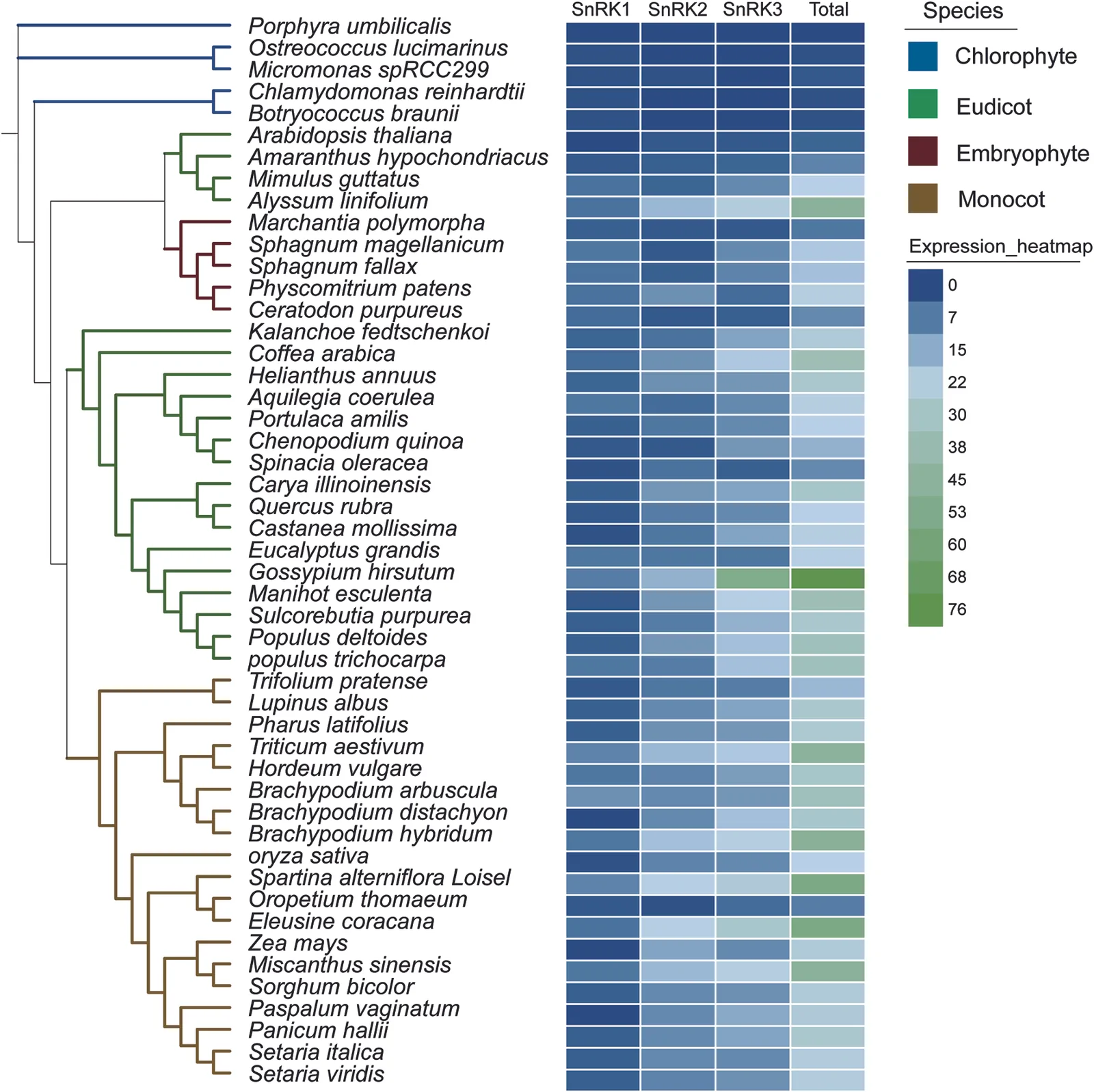
Identification of *SnRK* genes in 49 different species. The total number of identified *SnRK* genes is 1192, including 186 SnRK1, 409 SnRK2, and 597 SnRK3 genes. Gene copy numbers are shown for each species, with a focus on the evolutionary transition from algae to bryophytes, dicotyledons, and monocotyledons.

### 3.2 Identification of *SnRK* genes in *Spartina alterniflora*

Building on the genome-wide identification of *SnRK* genes across 49 species, we further characterized the *SaSnRK* gene family in the genome of *S. alterniflora*. Through sequence alignment, a total of 55 *SaSnRK* genes were identified at the genome level. Phylogenetic analysis with rice *SnRK* genes classified these 55 *SaSnRK* genes into three subfamilies, including *SaSnRK1* (9 genes), *SaSnRK2* (21 genes), and *SaSnRK3* (25 genes) (**Figures 2**, **3A**).

**Figure 2:**
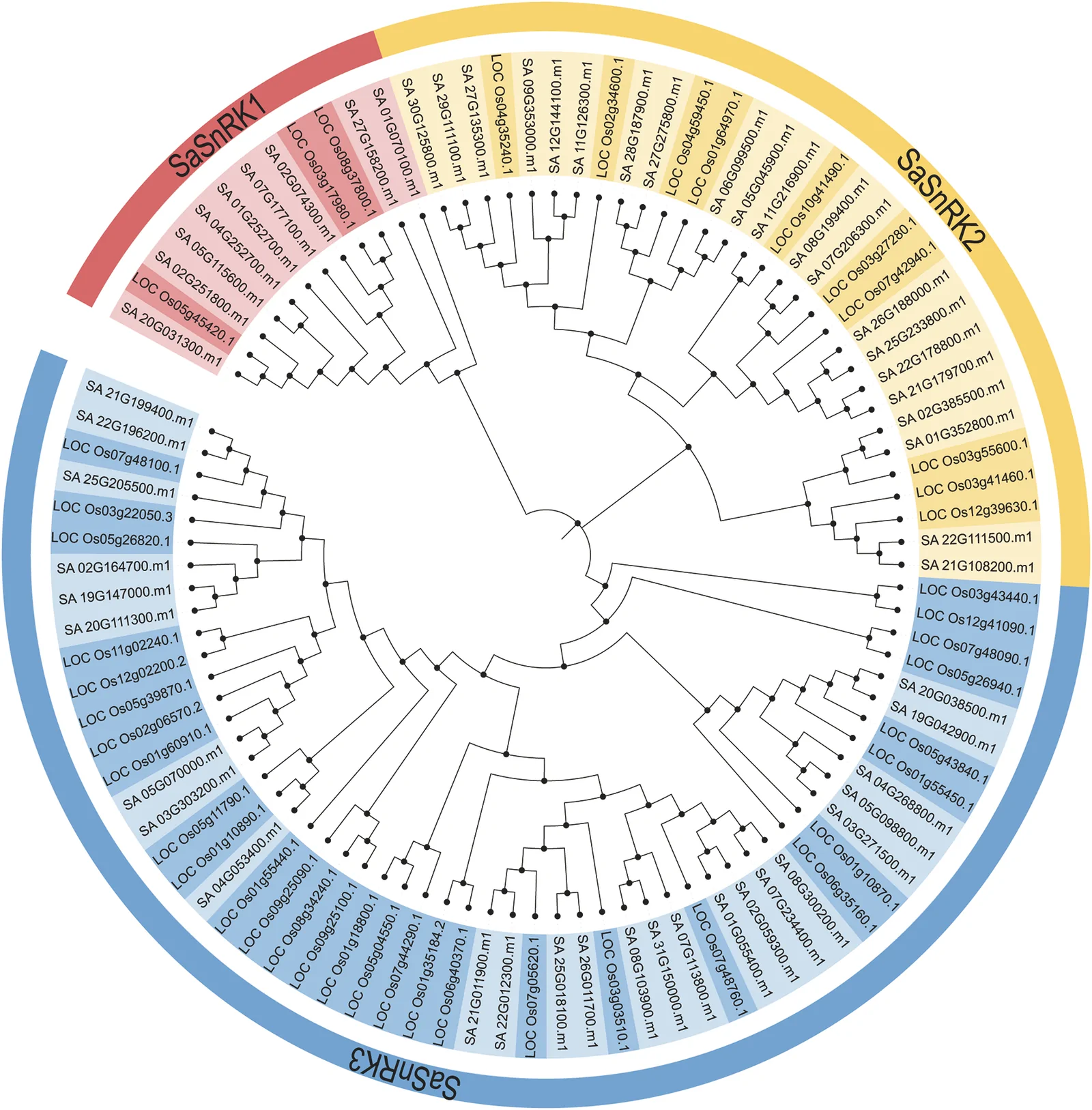
Phylogenetic analysis and classification of *SnRK* genes in *Spartina alterniflora*. A phylogenetic tree was constructed based on the amino acid sequences of SnRK proteins from *S. alterniflora* (*SaSnRK*) and rice (*OsSnRK*).

To gain further insight into the characteristics of the *SnRK* gene family, we analyzed the physicochemical properties of the SaSnRK members (**Supplementary Table S2**). These 55 members are unevenly distributed across 22 chromosomes, being absent only on chromosomes 10, 13–18, and 23–24. Physicochemical property analysis revealed that protein lengths ranged from 209 amino acids (SaSnRK1.9) to 2,602 amino acids (SaSnRK3.18), molecular weights ranged from 24.10 kDa (SaSnRK1.9) to 286.30 kDa (SaSnRK3.18), and isoelectric points ranged from 4.78 (SaSnRK2.2) to 9.66 (SaSnRK3.8). The instability index ranged from 29.27 (SaSnRK2.5) to 57.48 (SaSnRK1.7). Except for SaSnRK1.2, all SaSnRK1 members had instability indices above 40, indicating they are likely unstable proteins. In the SaSnRK2 subfamily, 11 genes had indices above 40 and 10 below 40, while in SaSnRK3 subfamily, 10 genes were above 40 and 15 below 40, suggesting that SaSnRK2 and SaSnRK3 subfamilies are generally more stable than SaSnRK1. The aliphatic index ranged from 73.29 (SaSnRK3.18) to 100.70 (SaSnRK1.2), and the grand average of hydropathicity (GRAVY) for all SaSnRK members was below 0, ranging from −0.001 (SaSnRK1.1) to –0.578 (SaSnRK3.8), indicating they are hydrophilic proteins.

### 3.3 Gene structure and motif analysis of *SaSnRK* genes

To investigate the structural characteristics of *SaSnRK* genes, we analyzed their gene structures and conserved protein motifs (**Figure 3**). In the SaSnRK1 subfamily, except for *Sa_01G252700*, most genes contained 9 to 12 exons and 1 to 2 UTRs. In the SaSnRK2 subfamily, except for *SaSnRK2.4*, *SaSnRK2.7*, and *SaSnRK2.18*, most genes had 8 to 9 exons and 1 to 2 UTRs. For the SaSnRK3 subfamily, genes could be divided into two categories. The first category contained 1 to 5 exons and 0 to 3 UTRs, with fewer but longer coding sequences. The second category contained 14 to 23 exons and 0 to 3 UTRs, with more numerous but shorter exons (**Figure 3B**). Motif analysis further revealed subfamily-specific and shared patterns (**Figure 3C**). Motifs 11 and 12 are unique to the SaSnRK1 subfamily, while motifs 6, 10, 13, and 15 are exclusively present in SaSnRK3 members, with some SaSnRK3 members additionally carrying motif 14 at the C-terminal. Notably, a core motif combination (motifs 5, 3, 2, 8, 1, 7, 4, and 9) is present in all SaSnRK proteins except *SaSnRK1.2*. Within the SaSnRK2 subfamily, motifs 1–8 consist solely of this core set, and some members also possess motif 14 appended at the C-terminal. This observation suggests that the SaSnRK2 subfamily is structurally more conserved than SaSnRK1 and SaSnRK3.

**Figure 3:**
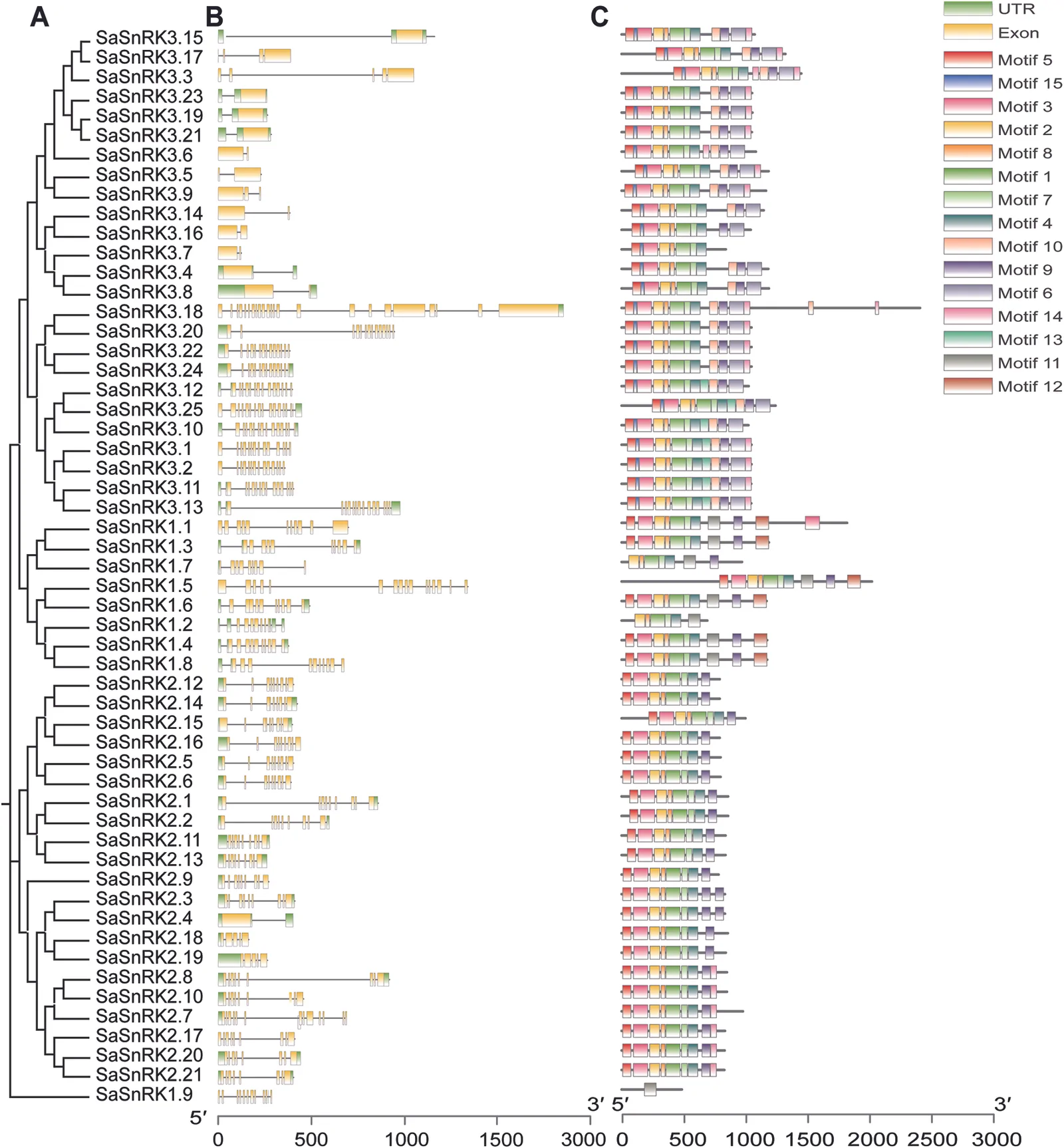
Phylogenetic relationships, gene structures, and conserved motifs of *SaSnRK* genes. **(A)** Phylogenetic tree of 55 SaSnRK proteins from *S. alterniflora* constructed using the neighbor-joining method with 1,000 bootstrap replicates, with exon–intron structures shown to the right **(B)**. Yellow boxes represent exons (CDS), green boxes indicate untranslated regions (UTRs), and black lines represent introns. **(C)** Conserved motif compositions of SaSnRK proteins. Fifteen distinct motifs (1–15) are represented by colored boxes, and their relative positions within each protein are shown.

### 3.4 Synteny analysis of *SaSnRK* genes

To further investigate the evolutionary relationships between *SaSnRK* genes and their counterpart in other species, we performed synteny analysis with rice, maize, and *Arabidopsis*. Intraspecific synteny analysis revealed 70 syntenic gene pairs involving 55 *SaSnRK* genes within the *S. alterniflora* genome. Of these, 64 pairs resulted from segmental duplication events, whereas the remaining 6 pairs wree identified as tandem duplications (**Figure 4A**; **Supplementary Table S3**). Interspecific synteny analysis showed that *SaSnRK* genes shared 24 syntenic gene pairs with maize (**Figure 4B**; **Supplementary Table S4**), 20 with rice (**Figure 4C**; **Supplementary Table S5**), and only 4 with *Arabidopsis* (**Figure 4D**; **Supplementary Table S6**). Among the three subfamilies, *SaSnRK3* exhibited the highest number of syntenic pairs with both maize and rice.

**Figure 4:**
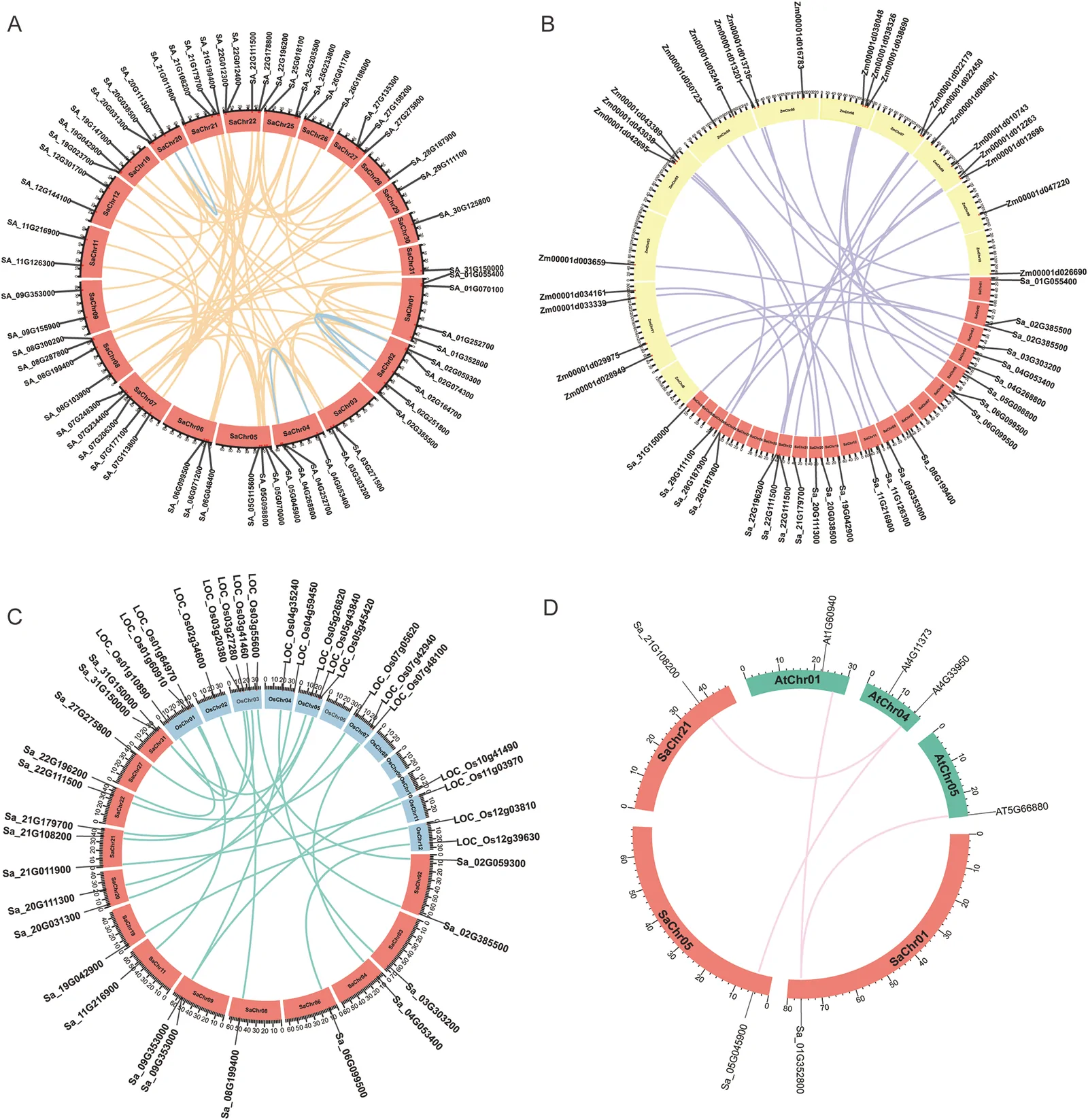
Synteny analysis of *SaSnRK* genes. **(A)** Intraspecific synteny of *SaSnRK* genes within the *S. alterniflora* genome is shown, including 6 tandem duplication events (blue lilnes) and 64 segmental duplication events (yellow lines). Interspecific synteny between *SaSnRK* genes and those of maize **(B)**, rice **(C)**, and *A. thaliana* **(D)** is also presented.

### 3.5 Mutation pressure response in *SaSnRK* genes

Given that the first and second positions of codons typically determine the amino acid type, while the third position is often synonymous, plants tend to maintain stability in GC1 and GC2 content during evolution to preserve gene function, whereas GC3 is less subject to natural selection. Within the *SaSnRK* gene family, the slopes for the *SaSnRK1*, *SaSnRK2*, and *SaSnRK3* subfamilies were 0.2328, 0.3714, and 0.654, respectively, with corresponding R² values of 0.4885, 0.3559, and 0.6146 (**Figures 5A–C**). However, these R² values are relatively low and the sample sizes within each subfamily are limited. The data suggest that the *SaSnRK3* subfamily, characterized by a higher slope and larger R² value, may be more susceptible to evolutionary pressure, whereas *SaSnRK1* and *SaSnRK2* appear relatively stable and more conserved during evolution. Furthermore, the *SaSnRK2* subfamily predominantly consists of genes with high GC3s and low ENC values (**Figures 5D–F**), indicating relatively strong codon usage bias. High GC content is known to enhance translation efficiency and accuracy, leading to the inference that SaSnRK2 gene family members may largely represent highly expressed genes.

**Figure 5:**
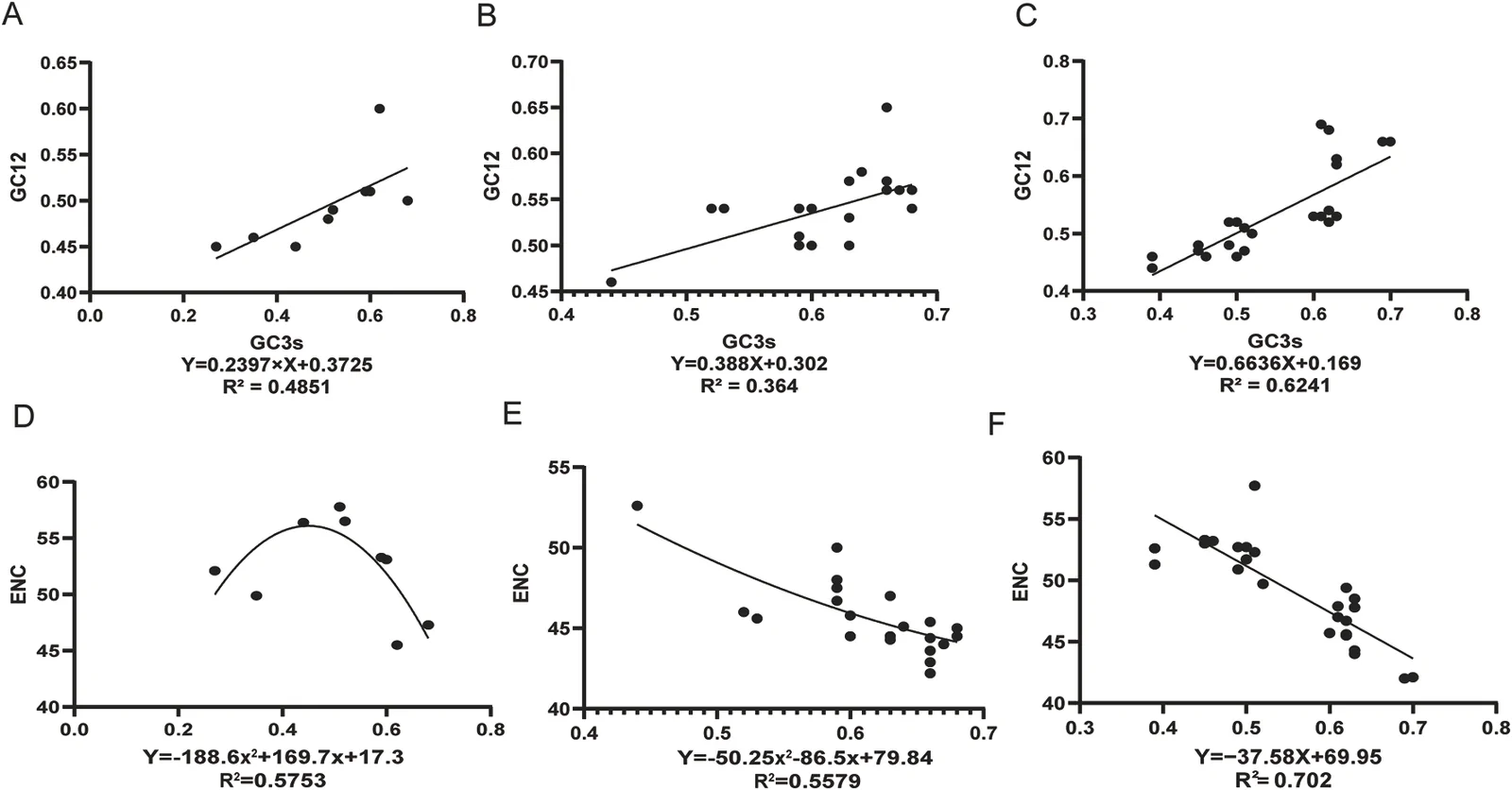
Analysis of the evolutionary mechanisms of *SaSnRK* genes. **(A–C)** GC3 versus GC12 scatter plots for SnRK1 **(A)**, SnRK2 **(B)**, and SnRK3 **(C)** subfamilies, respectively. **(D–F)** GC3 versus ENC scatter plots for SnRK1 **(D)**, SnRK2 **(E)**, and SnRK3 **(F)** subfamilies, respectively.

### 3.6 *Cis*-acting element analysis

To investigate the potential regulatory mechanisms of *SaSnRK* genes, *cis*-acting elements within 2-kb promoter regions upstream of the transcription start site (TSS) were predicted. The identified motifs were primarily classified into three functional categories, abiotic stress responses, plant growth and development, and phytohormone responses (**Figure 6**; **Supplementary Table S7**). Abiotic stress-related elements mainly included light-responsive elements, MYB binding sites involved in drought inducibility, low-temperature responsive elements, and anaerobic induction motifs. Growth and development-associated elements encompassed motifs regulating endosperm expression, meristem expression, and palisade mesophyll cells. Furthermore, several phytohormone-responsive elements were highly enriched, notably those responsive to abscisic acid (ABA), methyl jasmonate (MeJA), gibberellin (GA), auxin (IAA), and salicylic acid (SA). Additionally, basal regulatory elements, such as DNA-binding proteins (ATBP-1) and core promoter elements (*e.g.*, TATA-box located around -30 bp), were also detected. Overall, the composition and abundance of these *cis*-elements varied considerably among individual *SaSnRK* genes, implying diverse and complex transcriptional regulatory networks.

**Figure 6:**
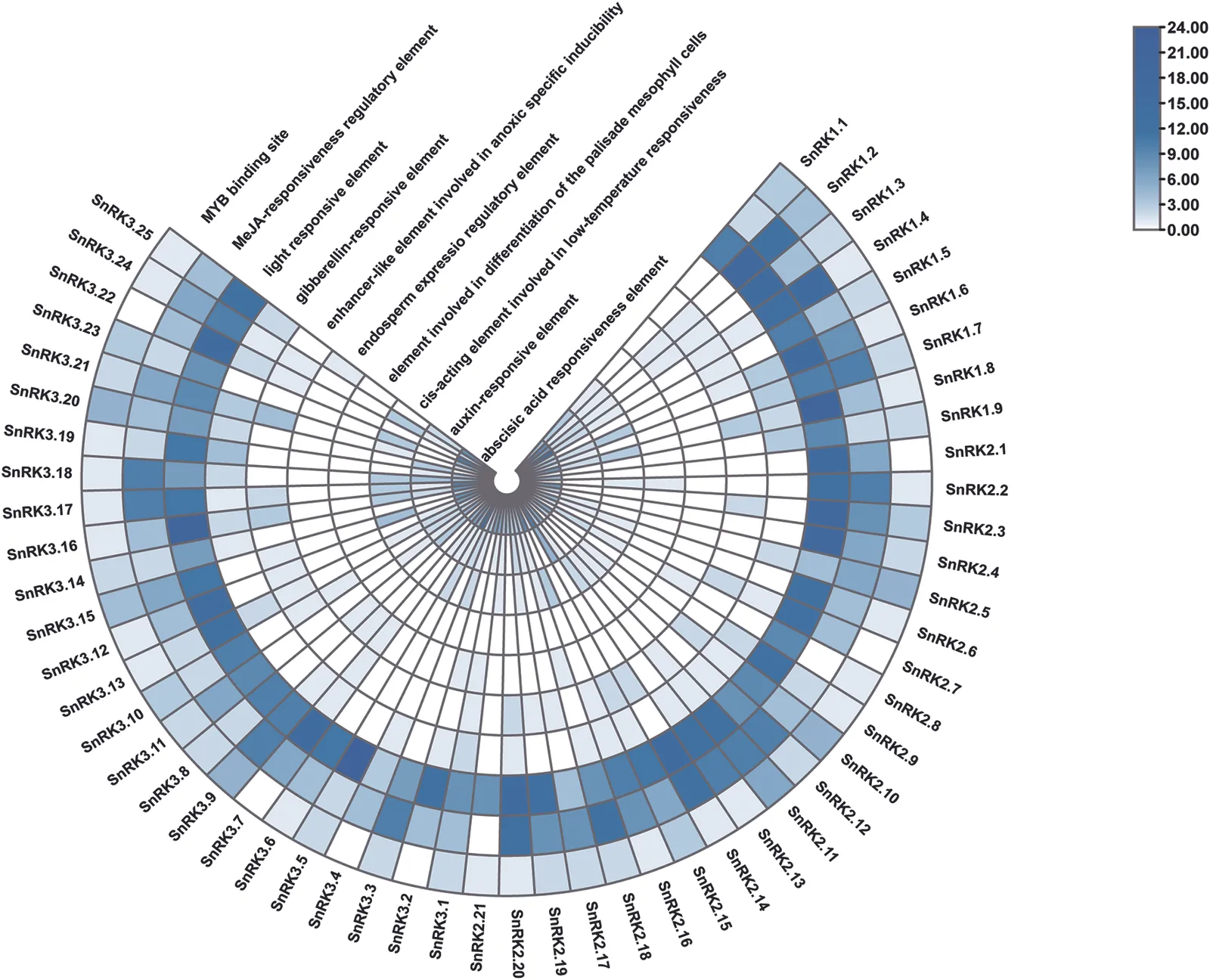
*Cis*-acting element analysis of *SaSnRK* gene promoters. The darker the color, the higher the number of elements.

### 3.7 Expression analysis of *SaSnRK* genes under salt stress

To explore the expression patterns of *SaSnRK* genes under salt stress, we analyzed their log2 fold changes at increasing NaCl concentrations (100–700 mM) relative to the control (0 mM). Within the SaSnRK2 subfamily, some genes were downregulated under low-salt conditions (100–300 mM) but became upregulated under high-salt stress (500–700 mM), whereas others were consistently upregulated across all concentration tested, (**Figure 7A**). For instance, *SaSnRK2.3* exhibited negative log_2_ fold changes at 100 mM (−0.17), 200 mM (−0.66), and 300 mM (−0.56), became positive at 500–700 mM, reaching 0.44 at 700 mM, fitting the first pattern. In contrast, *SaSnRK2.11* was consistently upregulated across all concentrations, with values increasing from 0.04 (100 mM) to 0.37 (700 mM), representing the second pattern. A similar contrasting pattern was observed between *SaSnRK2.9* and *SaSnRK2.12*. *SaSnRK2.9* was slightly downregulated at 200–300 mM but strongly induced at 500 mM (1.28), while *SaSnRK2.12* remained negative at all concentrations, with the lowest expression at 300 mM (−0.31) and 700 mM (−0.37). The qRT-PCR results for *SaSnRK2.9* were consistent with the transcriptome data, revealing significant upregulation under 100, 500, and 700 mM NaCl, with the highest transcript levels detected at 0.5 h post-treatment; in contrast, downregulation was observed under 200 and 300 mM NaCl (**Figure 7B**). No such clear pattern was observed in the SaSnRK1 or SaSnRK3 subfamilies, although individual members of both subfamilies showed notable upregulation under high salt conditions.

**Figure 7:**
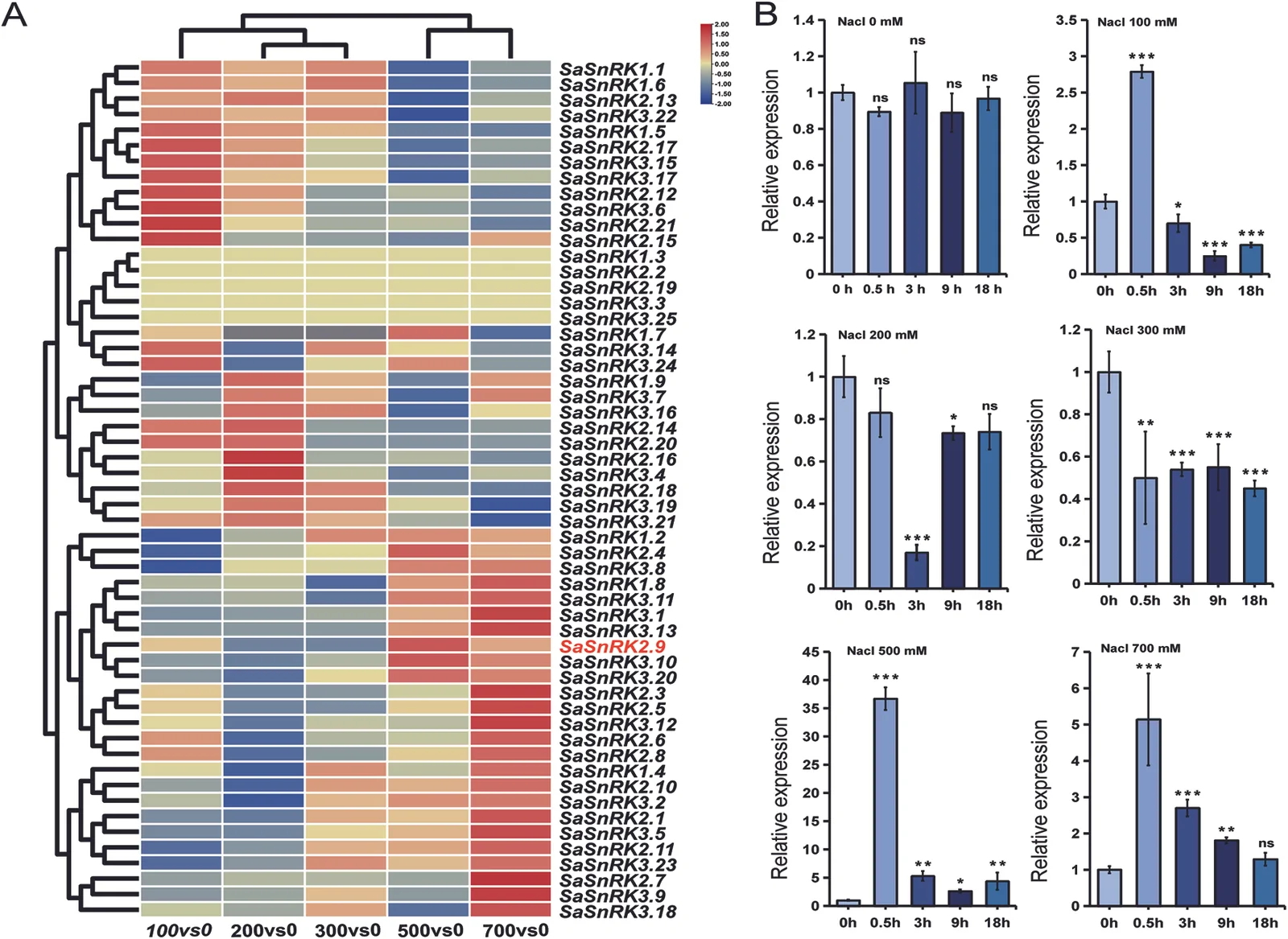
Expression patterns of *SaSnRK* genes under salt stress. **(A)** Log_2_ fold changes of SaSnRK1, SaSnRK2, and SaSnRK3 subfamily members under 100, 200, 300, 500, and 700 mM NaCl relative to the control (0 mM), visualized from the transcriptome dataset. Red indicates upregulation and blue indicates downregulation, with color intensity proportional to the magnitude of log2 fold change. The color scale ranges from −2 (blue) to +2 (red) as shown in the legend. Within each subfamily, genes are ordered by hierarchical clustering based on their expression similarity across salt concentrations. **(B)** qRT-PCR analysis of *SaSnRK2.9* expression over time (0–18 h) under salt treatments of 100, 200, 300, 500, and 700 mM, respectively. Values are depicted as mean ± SD (n = 3 biological replicates). The relative expression levels of the *SaSnRK2.9* genes were determined using the 2^−ΔΔC^T^^ method. * *p* < 0.05, ** *p* < 0.01, ***p < 0.001.

### 3.8 Functional characterization of *SaSnRK2.9*

The SaSnRK2 subfamily has a conserved evolutionary background and plays well-recognized roles in plant growth and development. *SaSnRK2.9*, which was initially identified as a core candidate through our previously established phylogenomic screening pipeline (Gao et al., 2023), was further found to be most strongly induced under 500 mM NaCl (**Figures 7A, B**). Therefore, we selected *SaSnRK2.9*, one of the two *SaSnRK2* genes most strongly induced at 500 mM NaCl, for further functional study. GUS reporter analysis in transgenic *Arabidopsis* revealed that the *SaSnRK2.9* promoter is predominantly active in seedling roots and mature siliques (**Figure 8A**), suggesting potential roles in root and silique development. Subcellular localization analysis indicated that the *SaSnRK2.9* is localized in the nucleus and cytoplasm (**Figure 8B**). To investigate the potential interaction network of *SaSnRK2.9*, the STRING database was used to predict candidate interacting protein. This analysis identified PP2C06, PP2C08, and PP2C30 as candidate interactors (**Figure 8C**). Through homology alignment, their corresponding gene names in *S. alterniflora* were determined as Sa_06G148900, Sa_02G086000, and Sa_08G277100, respectively. The results showed that SaSnRK2.9 specifically interacts with Sa_02G086000 and Sa_08G277100, whereas no interaction was detected with Sa_06G148900 (**Figure 8D**). These findings were further confirmed by LCI assays (**Figure 8E**).

**Figure 8:**
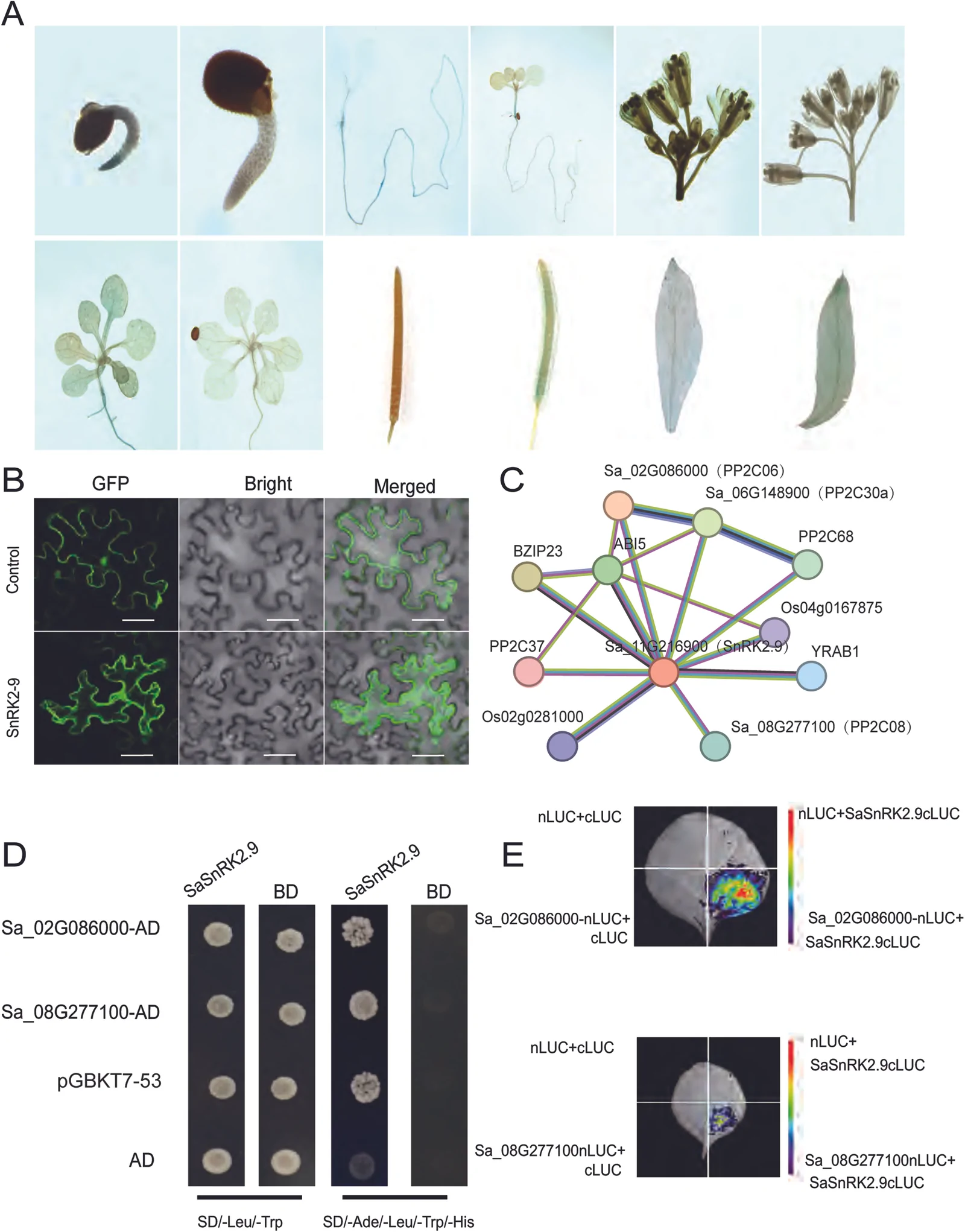
Expression pattern, subcellular localization, and protein interactions prediction of *SaSnRK2.9*. **(A)** GUS staining showing predominant expression in seedling roots and mature pods. **(B)** Subcellular localization indicating that *SaSnRK2.9* is mainly present in the nucleus and cytoplasm, scale bar = 2 μm. **(C)** STRING-predicted interaction network identifying Sa_02G086000 (PP2C06), Sa_08G277100 (PP2C08), and Sa_06G148900 (PP2C30) as candidate interactors. **(D)** Y2H assay results showing that *SaSnRK2.9* specifically interacts with *Sa_02G086000* and *Sa_08G277100*, but not with *Sa_06G148900*. **(E)** LCI assays confirmed that *SaSnRK2.9* specifically interacts with *Sa_02G086000* and *Sa_08G277100*.

### 3.9 Overexpression of *SaSnRK2.9* promotes early flowering and salt tolerance

To confirm that the observed phenotypes correlate with *SaSnRK2.9* expression, we first examined transgene transcript levels in multiple independent transgenic lines by qRT-PCR. Three lines (OE1, OE2, and OE3) with significantly elevated SaSnRK2.9 expression compared to wild-type plants (**Supplementary Figure S1**) were selected for subsequent phenotypic characterization. Further, we performed a comparative analysis between OE lines and wild-type plants. The results revealed that OE lines exhibited an advanced flowering time by 4–6 d relative to the wild type (**Figures 9A, B**). In addition, these lines produced more rosette leaves (14–16 compared to 12 in the wild type) and displayed a larger rosette radius (**Figures 9C–E**). More importantly, under salt stress, the OE lines showed significantly enhanced tolerance compared with the wild type (**Figure 9F**), with a survival rate exceeded 50% following a one-week recovery period, whereas that of the wild type was less than 20% (**Figure 9G**).

**Figure 9:**
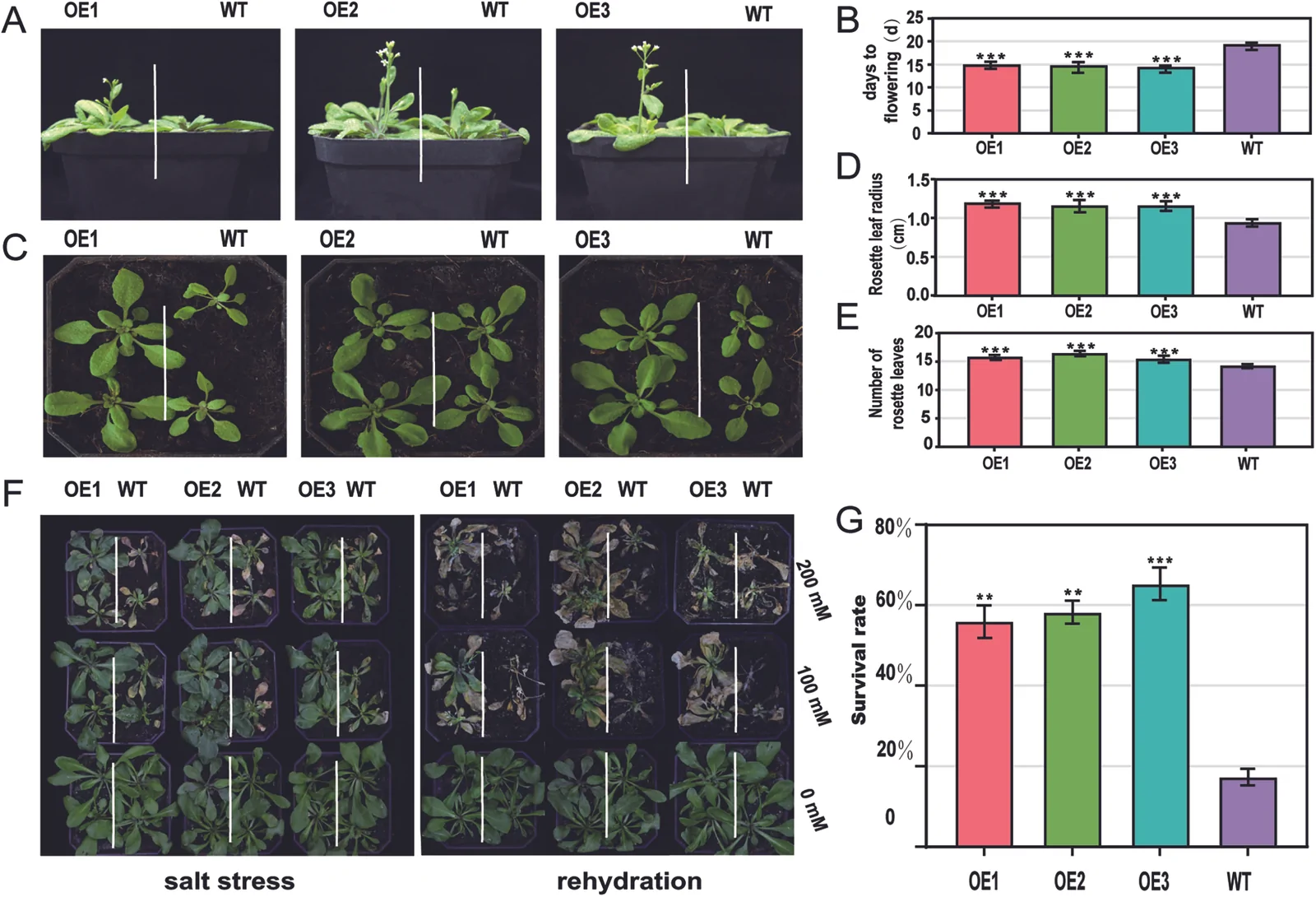
Phenotypic characterization of overexpression lines (OE1, OE2, OE3) and salt tolerance. **(A)** Representative images of plants at flowering time. **(B)** Flowering time measured as days from sowing to first flower opening. Data are presented as mean ± SE (n = 3 independent experiments, each with at least 10 plants per line). Statistical significance was determined by one-way ANOVA followed by Tukey’s post-hoc test (**P < 0.01, *P < 0.001). **(C)** Representative images of rosette leaves. **(D)** Rosette leaf number counted at the flowering stage. Data are mean ± SE (n = 3, ≥10 plants per line); **P < 0.01, *P < 0.001 (one-way ANOVA with Tukey’s post-hoc test). **(E)** Rosette radius measured as the longest distance from the center to the leaf tip. Data are mean ± SE (n = 3, ≥10 plants per line); **P < 0.01, *P < 0.001 (one-way ANOVA with Tukey’s post-hoc test). **(F)** Representative images of plant during salt treatment at the rosette leaf stage. **(G)** Survival rate of plants at the rosette leaves stage under salt treatment. Data in **(D, E)** and **(G)** are presented as mean ± SE. Data are presented as percentage survival (n = 3 independent experiments, each with ≥15 plants per line). Significance was assessed using Fisher’s exact test comparing each OE line to the wild type; **P < 0.01, *P < 0.001.

## 4 Discussion

### 4.1 Expansion and evolutionary dynamics of the *SaSnRK* gene family in *S. alterniflora*

The *SaSnRK* gene family has undergone remarkable expansion throughout the plant kingdom, and our genome-wide identification across 49 species provides a comprehensive perspective on this evolutionary trajectory. The progressive increase in *SaSnRK* copy numbers from algae to angiosperms strongly suggests that gene duplication events have been crucial in equipping plants with the regulatory complexity required to colonise diverse and often stressful terrestrial environments. Notably, the significant expansion observed in certain monocotyledons, including *S. alterniflora*, indicates a lineage-specific adaptive strategy. The 55 *SaSnRK* genes identified in *S. alterniflora* represent a substantial family size, comparable to the 60 *ZmSnRK*s reported in maize and exceeding the 38 found in *Arabidopsis* (Feng et al., 2022; Hrabak et al., 2003). This expansion is particularly evident in the *SnRK2* and *SnRK3* subfamilies, consistent with the notion that these plant-specific kinases are central to abiotic stress adaptation (Ai et al., 2022; Heinemann and Hildebrandt, 2021; Mondal et al., 2024).

Synteny analysis revealed that segmental duplications have been the primary drivers of *SaSnRK* family expansion, with 64 segmental duplication events compared to only six tandem duplications. The higher number of syntenic pairs shared with monocot species (24 with maize and 20 with rice) compared to the dicot *Arabidopsis* (only 4 pairs) reflects the closer evolutionary relationship between *S. alterniflora* and other monocots. This finding aligns with observations in *Brassica napus* and *Rosaceae* species, where whole-genome duplication (WGD) events also predominantly contributed to *SnRK* family proliferation (Wang et al., 2023; Zhu et al., 2020). The preferential retention of SaSnRK3 subfamily members in syntenic pairs with rice and maize is particularly noteworthy. Given that SaSnRK3 subfamily members in syntenic pairs with rice and maize is particularly noteworthy. Given that *SnRK3* (CIPK) genes function as calcium signal decoders in collaboration with CBL proteins (Li et al., 2023), their preferential retention following polyploidization may have conferred an enhanced capacity to respond to a wide array of environmental cues, a clear advantage for a halophyte constantly exposed to fluctuating salinity.

Codon usage analysis further differentiates the three subfamilies. The higher slope and R² value observed in the SaSnRK3 subfamily in the neutrality plot suggest that pressure has played a greater role in shaping its codon usage, possibly reflecting its diversification to accommodate diverse stress signals. Conversely, the SaSnRK2 subfamily, characterized by lower ENC values and higher GC3 content, appears to be under stronger selective constraints that favor translation efficiency and accuracy. This codon usage pattern, together with the constitutive expression of several *SaSnRK2* members observed in our transcriptome data, suggests that at least some *SaSnRK2* genes may maintain relatively stable expression levels, consistent with their well-established roles as core components of ABA signaling that require rapid and precise activation (Hasan et al., 2022; Nakashima and Yamaguchi-Shinozaki, 2013). Nevertheless, we acknowledge that these inferences remain indirect and would benefit from additional independent expression or translational efficiency assays. In contrast, the instability indices suggest that SaSnRK1 proteins may be more short-lived or tightly regulated post-translationally, aligning with their function as energy sensors that need to respond dynamically to metabolic fluctuations (Blanford et al., 2024; Wang et al., 2022).

### 4.2 Divergent salt-responsive expression patterns and regulatory complexity

The expression profiles of *SaSnRK* genes under increasing salt concentrations reveal two contrasting behaviors within the SaSnRK2 subfamily, one is genes were induced only at high salinity (*e.g*., *SaSnRK2.3*), while others showed constitutive upregulated across all test concentrations (*e.g*., *SaSnRK2.11*). This sort of dichotomy has been observed in other species. For example, in wheat, *TaSnRK2.4* and *TaSnRK2.8* are induced by multiple stresses, whereas other members exhibit more specific responses (Feng et al., 2018; Jiang et al., 2022; Zhang et al., 2017). In rice, *OsSAPK8* is broadly stress-responsive, while other *SAPK* members respond more selectively (Zhong et al., 2020). The expression patterns observed in *S. alterniflora* suggest the coexistence of at least two functional classes of *SaSnRK2* genes: those maintaining a basal level of stress readiness across a wide range, and those recruited primarily under severe salinity. This distinction may reflect a regulatory strategy whereby certain isoforms maintain a basal level of stress readiness, while others are recruited primarily under extreme conditions, potentially minimizing unnecessary growth diversion under mild stress. If confirmed by further functional studies, this pattern could represent a molecular mechanism contributing to the remarkable salt tolerance of this halophyte.

The absence of clear salt-responsive patterns in the SaSnRK1 and SaSnRK3 subfamilies does not imply functional irrelevance, rather, it likely reflects their involvement in more indirect or context-dependent stress responses. *SnRK1* is activated by the secondary metabolic consequences of salt stress (Wang et al., 2022), while *SnRK3* expression varies depending on its CBL partners and cell type (Li et al., 2023). This variability is consistent with the rich diversity of *cis*-acting elements, including ABRE, MeJA-responsive, and auxin-responsive elements were identified in their promoters, suggesting their integration into multiple signaling hubs.

GUS reporter analysis in transgenic *Arabidopsis* indicated that the *SaSnRK2.9* promoter is active predominantly in roots and mature siliques, suggesting that the regulatory elements driving tissue-specific expression may be conserved across species. However, direct verification of the endogenous expression pattern in *S. alterniflora* tissues will be required to confirm this inference. STRING-based protein interaction network prediction identified PP2C proteins *Sa_02G086000*, *Sa_08G277100*, and *Sa_06G148900* as candidate interactors of *SaSnRK2.9*. The predicted association with PP2C proteins is consistent with the well-established ABA signaling module, in which PP2Cs normally repress SnRK2 activity until displaced by ABA-bound PYR/PYL receptors (Hasan et al., 2022), suggesting that *SaSnRK2.9* may function within this canonical pathway. This predicted interaction was experimentally confirmed by yeast two-hybrid and LCI assays, which demonstrated that *SaSnRK2.9* specifically interacts with *Sa_02G086000* and *Sa_08G277100*, but not with *Sa_06G148900*. Taken together, these results place *SaSnRK2.9* within the ABA-dependent SnRK2-PP2C regulatory module, and support its role as a salt-inducible member that may contribute to the stress-responsive arm of the SnRK2 functional dichotomy observed in *S. alterniflora.*

### 4.3 Phenotypic consequences of *SaSnRK2.9* gene overexpression and implications for crop improvement

The phenotypic effects of overexpressing *SaSnRK2.9* in *Arabidopsis* are both striking and somewhat pleiotropic. Enhanced salt tolerance, as evidenced by a survival rate exceeding 50% in overexpression lines compared to less than 20% in wild-type plants following the one-week recovery period, confirms the functional role of this gene in stress adaptation. In addition, overexpression lines displayed early flowering, increasing rosette leaf number, and a larger rosette radius relative to wild-type controls, suggesting that *SaSnRK2.9* promotes overall growth vigor rather than merely conferring stress protection. This is noteworthy because *SnRK2*s are canonically associated with stress-induced growth inhibition mediated through ABA signaling (Li et al., 2025; Lozano-Juste et al., 2020). However, our findings are consistent with emerging evidence that certain SnRK2 isoforms can simultaneously promote stress tolerance and growth. For example, *SmSnRK2.6* overexpression in *Salvia miltiorrhiza* enhanced stress tolerance without comprising growth or secondary metabolism (Liu et al., 2024), and *HcSnRK2.2* overexpression in *Hedychium coronarium* promoted floral development (Wang et al., 2021). However, our study provides the first experimental evidence for such a dual role in a halophytic monocot, and the identification of two specific PP2C interactors (Sa_02G086000 and Sa_08G277100) as novel partners of SaSnRK2.9 further distinguishes this work from previous reports. These parallels suggest that the growth-promoting and stress-protective functions of SaSnRK2 proteins are not mutually exclusive, possibly reflecting the activation of distinct downstream targets in a tissue- or developmental stage-specific manner.

Building on these phenotypic observations, we propose two non-mutually exclusive hypotheses to explain the possible basis of the dual functionality, which warrant testing in future studies, particularly using kinase-dead mutants or CRISPR/Cas9-edited lines in *S. alterniflora*. First, *SaSnRK2.9* might phosphorylate a specific set of downstream targets that differ from those targeted by stress-inhibitory SnRK2s, thereby uncoupling stress signaling from growth suppression. Second, the early flowering phenotype could represent an adaptive “escape” strategy, allowing the plant to complete reproduction before salt stress becomes lethal. This interpretation is consistent with the tissue-specific expression of *SaSnRK2.9* in roots and siliques, which aligns with its proposed roles in both stress perception at the root–soil interface and reproductive development. Our protein interaction data further refine these models by placing *SaSnRK2.9* within a specific PP2C-regulated network. The selective interaction with Sa_02G086000 and Sa_08G277100, but not with Sa_06G148900, suggests that *SaSnRK2.9* is not a general ABA-signaling component but rather a specialized kinase that engages with a subset of PP2C partners. This specificity may underlie its unique functional output, distinguishing it from other SaSnRK2 members that interact more broadly with PP2Cs. Collectively, these findings advance our understanding of how SnRK2 kinases achieve functional diversification and provide a molecular framework for engineering crops that maintain yield under saline conditions.

## 5 Conclusion

In this study, we combined genome-wide identification with functional validation to dissect the *SaSnRK* gene family in the halophyte *Spartina alterniflora*. Through phylogenomic screening and experimental characterization, we demonstrate that *SaSnRK2.9* physically interacts with two specific PP2C proteins and, when overexpressed in *Arabidopsis*, confers enhanced salt tolerance while simultaneously promoting earlier flowering and increased vegetative growth. Although these phenotypic effects have not yet been directly validated in *S. alterniflora*, our results indicate that *SaSnRK2.9* has the potential to uncouple stress tolerance from growth suppression. This dual functionality challenges the prevailing view that SnRK2-mediated stress signaling inherently suppresses growth, and instead suggests that certain halophytic SnRK2 isoforms can couple stress adaptation with developmental acceleration. Our findings provide a working framework for the functional diversification of SnRK2 kinases and position *SaSnRK2.9* as a promising target for engineering both stress resilience and agronomic traits in crops. This study also underscores the value of halophytes as reservoirs of stress-adaptive regulatory modules with untapped potential for crop engineering.

## Data Availability

The original contributions presented in the study are included in the article/**Supplementary Material**. Further inquiries can be directed to the corresponding authors.
